# Supplementary material for: TPGPred: A Mixed-Feature-Driven Approach for Identifying Thermophilic Proteins Based on GradientBoosting
Source: Int J Mol Sci. 2024 Nov 5;25(22):11866. doi: 10.3390/ijms252211866 (PMC11594102; doi:10.3390/ijms252211866)
Supplement: Supplementary file 1 [file ijms-25-11866-s001.zip › ijms-3278932-supplementary.pdf]

Table_S 1 Comparative analysis of different machine learning algorithms and corresponding string feature engineering methods (Supplement 3 Methods).

	Recall	Precision	Accuracy	F1 Score	MCC	Specificity	AUROC	AUPRC
DecisionTree_CountVectorizer	0.987755	0.401327	0.419458	0.570755	0.108661	0.054974	0.525745	0.404947
DecisionTree_CountVectorizer_n_grams	0.832653	0.41129	0.4689	0.550607	0.081917	0.235602	0.466236	0.381821
DecisionTree_HashingVectorizer	0.816327	0.749064	0.821372	0.78125	0.632443	0.824607	0.834795	0.714165
DecisionTree_LDA	0.865306	0.590529	0.712919	0.701987	0.473897	0.615183	0.888444	0.839775
DecisionTree_LSA	0.918367	0.526932	0.645933	0.669643	0.407824	0.471204	0.644396	0.496331
DecisionTree_PCA	0.738776	0.628472	0.727273	0.679174	0.449076	0.719895	0.831371	0.748296
DecisionTree_t-SNE	0.897959	0.554156	0.677831	0.685358	0.440001	0.536649	0.571578	0.492307
DecisionTree_Word2Vec	0.869388	0.891213	0.907496	0.880165	0.805024	0.931937	0.927663	0.890538
DecisionTree_FastText	0.812245	0.548209	0.665072	0.654605	0.37842	0.570681	0.515456	0.460038
DecisionTree_Doc2Vec	0.938776	0.555556	0.682616	0.698027	0.470908	0.518325	0.931291	0.903253
DecisionTree_BERT	0.987755	0.403333	0.424242	0.572781	0.121578	0.062827	0.527076	0.405614
MLPClassifier_CountVectorizer	0.869388	0.763441	0.8437	0.812977	0.683934	0.827225	0.906165	0.906734
MLPClassifier_CountVectorizer_n_grams	1	0.392	0.393939	0.563218	0.045303	0.005236	0.957955	0.961477
MLPClassifier_HashingVectorizer	0.902041	0.465263	0.556619	0.613889	0.269969	0.335079	0.839117	0.852132
MLPClassifier_LDA	0.983673	0.395082	0.405104	0.563743	0.053189	0.034031	0.658591	0.489612
MLPClassifier_LSA	0.942857	0.508811	0.62201	0.660944	0.39198	0.41623	0.90484	0.905651
MLPClassifier_PCA	0.971429	0.392092	0.400319	0.558685	0.01516	0.034031	0.560295	0.423654
MLPClassifier_t-SNE	0.983673	0.389968	0.392344	0.558517	-0.01328	0.013089	0.501325	0.391428
MLPClassifier_Word2Vec	1	0.392628	0.395534	0.563867	0.055529	0.007853	0.505684	0.393489
MLPClassifier_FastText	0.122449	1	0.657097	0.218182	0.279912	1	0.906058	0.916622
MLPClassifier_Doc2Vec	1	0.39075	0.39075	0.561927	0	0	0.494487	0.388125
MLPClassifier_BERT	0.987755	0.389694	0.39075	0.558891	-0.02201	0.007853	0.496153	0.388922
BiLSTM_CountVectorizer	1	0.395161	0.401914	0.566474	0.085095	0.018325	0.889529	0.86802
BiLSTM_CountVectorizer_n_grams	0.816327	0.806452	0.851675	0.811359	0.689194	0.874346	0.903943	0.901524
BiLSTM_HashingVectorizer	0.971429	0.390164	0.395534	0.556725	-0.00719	0.026178	0.829373	0.841716
BiLSTM_LDA	0.983673	0.391234	0.395534	0.559814	0.007426	0.018325	0.831184	0.845824
BiLSTM_LSA	0.987755	0.390953	0.393939	0.560185	0.00367	0.013089	0.831243	0.843758
BiLSTM_PCA	1	0.39075	0.39075	0.561927	0	0	0.850347	0.849119
BiLSTM_t-SNE	0.983673	0.393148	0.400319	0.561772	0.032533	0.026178	0.844364	0.851342
BiLSTM_Word2Vec	0.979592	0.394737	0.405104	0.56272	0.046227	0.036649	0.861257	0.851924
BiLSTM_FastText	0.991837	0.391935	0.395534	0.56185	0.022874	0.013089	0.876087	0.879028
BiLSTM_Doc2Vec	0.983673	0.392508	0.398724	0.561118	0.02477	0.02356	0.862838	0.87147
BiLSTM_BERT	1	0.39075	0.39075	0.561927	0	0	0.77676	0.795716

Since the four methods are not effective, they are not discussed in this article.

Table_S 2 Performance analysis of the Bagging_Doc2Vec model after removing the corresponding protein descriptors.

	Recall	Precision	Accuracy	F1 Score	MCC	Specificity	AUROC	AUPRC
AAC_Bagging_Doc2Vec	0.885714	0.753472	0.842105	0.814259	0.68521	0.814136	0.926269	0.908745

CKSAAGP_Bagging_Doc2Vec	0.836735	0.716783	0.807018	0.772128	0.611957	0.787958	0.895667	0.875362
CKSAAP_Bagging_Doc2Vec	0.853061	0.794677	0.856459	0.822835	0.703693	0.858639	0.927145	0.910231
CTDC_Bagging_Doc2Vec	0.869388	0.678344	0.787879	0.762075	0.590372	0.735602	0.888273	0.872757
CTDD_Bagging_Doc2Vec	0.918367	0.639205	0.76555	0.753769	0.57611	0.667539	0.92816	0.913063
CTDT_Bagging_Doc2Vec	0.738776	0.79386	0.822967	0.765328	0.624536	0.876963	0.903141	0.864392
CTriad_Bagging_Doc2Vec	0.906122	0.704762	0.814992	0.792857	0.646662	0.756545	0.931184	0.921329
DDE_Bagging_Doc2Vec	0.955102	0.592405	0.725678	0.73125	0.539284	0.578534	0.909515	0.870299
DPC_Bagging_Doc2Vec	0.893878	0.734899	0.832536	0.80663	0.67129	0.793194	0.933497	0.913934
GAAC_Bagging_Doc2Vec	0.62449	0.974522	0.84689	0.761194	0.691508	0.989529	0.949738	0.933288
GDPC_Bagging_Doc2Vec	0.8	0.816667	0.851675	0.808247	0.687429	0.884817	0.912608	0.888921
GTPC_Bagging_Doc2Vec	0.84898	0.787879	0.851675	0.817289	0.694118	0.853403	0.928288	0.915937
KSCTriad_Bagging_Doc2Vec	0.934694	0.581218	0.711324	0.716745	0.507629	0.568063	0.91739	0.897486
TPC_Bagging_Doc2Vec	0.195918	0.631579	0.641148	0.299065	0.183313	0.926702	0.536826	0.509512

Table_S 3 Difference in performance metrics between the Bagging_Doc2Vec model with individual protein descriptors removed and the baseline results. Baseline metrics were calculated using the full set of protein descriptors.

	Recall	Precision	Accuracy	F1 Score	MCC	Specificity	AUROC	AUPRC
AAC_Bagging_Doc2Vec	0.020408	-0.1564	-0.07177	-0.07277	-0.13298	-0.13089	-0.0238	-0.0382
CKSAAGP_Bagging_Doc2Vec	-0.02857	-0.19309	-0.10686	-0.1149	-0.20623	-0.15707	-0.0544	-0.07158
CKSAAP_Bagging_Doc2Vec	-0.01224	-0.11519	-0.05742	-0.06419	-0.11449	-0.08639	-0.02292	-0.03671
CTDC_Bagging_Doc2Vec	0.004082	-0.23153	-0.126	-0.12495	-0.22781	-0.20942	-0.06179	-0.07419
CTDD_Bagging_Doc2Vec	0.053061	-0.27067	-0.14833	-0.13326	-0.24208	-0.27749	-0.0219	-0.03388
CTDT_Bagging_Doc2Vec	-0.12653	-0.11601	-0.09091	-0.1217	-0.19365	-0.06806	-0.04692	-0.08255
CTriad_Bagging_Doc2Vec	0.040816	-0.20511	-0.09888	-0.09417	-0.17152	-0.18848	-0.01888	-0.02561
DDE_Bagging_Doc2Vec	0.089796	-0.31747	-0.1882	-0.15578	-0.2789	-0.36649	-0.04055	-0.07664
DPC_Bagging_Doc2Vec	0.028571	-0.17497	-0.08134	-0.0804	-0.1469	-0.15183	-0.01657	-0.03301
GAAC_Bagging_Doc2Vec	-0.24082	0.064651	-0.06699	-0.12584	-0.12668	0.044503	-0.00033	-0.01366
GDPC_Bagging_Doc2Vec	-0.06531	-0.0932	-0.0622	-0.07878	-0.13076	-0.06021	-0.03746	-0.05802
GTPC_Bagging_Doc2Vec	-0.01633	-0.12199	-0.0622	-0.06974	-0.12407	-0.09162	-0.02178	-0.03101
KSCTriad_Bagging_Doc2Vec	0.069388	-0.32865	-0.20255	-0.17028	-0.31056	-0.37696	-0.03267	-0.04946
TPC_Bagging_Doc2Vec	-0.66939	-0.27829	-0.27273	-0.58796	-0.63487	-0.01832	-0.41324	-0.43743

Table_S 4 Performance Analysis of the RandomForest_Doc2Vec Model after the Removal of Protein Descriptors

	Recall	Precision	Accuracy	F1 Score	MCC	Specificity	AUROC	AUPRC
AAC_RandomForest_Doc2Vec	0.857143	0.9375	0.92185	0.895522	0.835436	0.963351	0.958756	0.952987
CKSAAGP_RandomForest_Doc2Vec	0.840816	0.899563	0.901116	0.869198	0.791021	0.939791	0.943498	0.942804
CKSAAP_RandomForest_Doc2Vec	0.836735	0.887446	0.894737	0.861345	0.777503	0.931937	0.944877	0.938287
CTDC_RandomForest_Doc2Vec	0.893878	0.820225	0.881978	0.855469	0.758045	0.874346	0.959461	0.952416
CTDD_RandomForest_Doc2Vec	0.902041	0.840304	0.894737	0.870079	0.783182	0.890052	0.949428	0.94294
CTDT_RandomForest_Doc2Vec	0.791837	0.960396	0.905901	0.868009	0.804895	0.979058	0.950732	0.948808
CTriad_RandomForest_Doc2Vec	0.869388	0.848606	0.888357	0.858871	0.766699	0.900524	0.945753	0.94354
DDE_RandomForest_Doc2Vec	0.836735	0.923423	0.909091	0.877944	0.808282	0.955497	0.953638	0.951862

DPC_RandomForest_Doc2Vec	0.840816	0.907489	0.904306	0.872881	0.797824	0.945026	0.960989	0.952477
GAAC_RandomForest_Doc2Vec	0.865306	0.909871	0.913876	0.887029	0.818186	0.945026	0.957218	0.953269
GDPC_RandomForest_Doc2Vec	0.885714	0.864542	0.901116	0.875	0.793384	0.910995	0.959301	0.951218
GTPC_RandomForest_Doc2Vec	0.889796	0.848249	0.894737	0.868526	0.781464	0.897906	0.953745	0.951168
KSCTriad_RandomForest_Doc2Vec	0.84898	0.866667	0.889952	0.857732	0.768129	0.91623	0.940784	0.928357
TPC_RandomForest_Doc2Vec	0.873469	0.737931	0.829346	0.8	0.660075	0.801047	0.929458	0.921561

Table_S 5 Performance Metrics and Differences from Baseline after Stepwise Removal of Protein Descriptors in the RandomForest_Doc2Vec Model

	Recall	Precision	Accuracy	F1 Score	MCC	Specificity	AUROC	AUPRC
AAC_RandomForest_Doc2Vec	-0.01633	0.042103	0.011164	0.011225	0.023683	0.028796	0.000705	0.000745
CKSAAGP_RandomForest_Doc2Vec	-0.03265	0.004166	-0.00957	-0.0151	-0.02073	0.005236	-0.01455	-0.00944
CKSAAP_RandomForest_Doc2Vec	-0.03673	-0.00795	-0.01595	-0.02295	-0.03425	-0.00262	-0.01317	-0.01395
CTDC_RandomForest_Doc2Vec	0.020408	-0.07517	-0.02871	-0.02883	-0.05371	-0.06021	0.00141	0.000174
CTDD_RandomForest_Doc2Vec	0.028571	-0.05509	-0.01595	-0.01422	-0.02857	-0.0445	-0.00862	-0.0093
CTDT_RandomForest_Doc2Vec	-0.08163	0.064999	-0.00478	-0.01629	-0.00686	0.044503	-0.00732	-0.00343
CTriad_RandomForest_Doc2Vec	-0.00408	-0.04679	-0.02233	-0.02543	-0.04506	-0.03403	-0.0123	-0.0087
DDE_RandomForest_Doc2Vec	-0.03673	0.028026	-0.00159	-0.00635	-0.00347	0.020942	-0.00441	-0.00038
DPC_RandomForest_Doc2Vec	-0.03265	0.012091	-0.00638	-0.01142	-0.01393	0.010471	0.002938	0.000235
GAAC_RandomForest_Doc2Vec	-0.00816	0.014474	0.00319	0.002732	0.006432	0.010471	-0.00083	0.001027
GDPC_RandomForest_Doc2Vec	0.012245	-0.03086	-0.00957	-0.0093	-0.01837	-0.02356	0.00125	-0.00102
GTPC_RandomForest_Doc2Vec	0.016327	-0.04715	-0.01595	-0.01577	-0.03029	-0.03665	-0.00431	-0.00107
KSCTriad_RandomForest_Doc2Vec	-0.02449	-0.02873	-0.02073	-0.02657	-0.04362	-0.01832	-0.01727	-0.02389
TPC_RandomForest_Doc2Vec	-2.4E-10	-0.15747	-0.08134	-0.0843	-0.15168	-0.13351	-0.02859	-0.03068

Table_S 6 Performance Analysis of the GradientBoosting_Word2Vec Model after the Removal of Protein Descriptors

	Recall	Precision	Accuracy	F1 Score	MCC	Specificity	AUROC	AUPRC
AAC_GradientBoosting_HashingVectorizer	0.853061	0.963134	0.929825	0.904762	0.85345	0.979058	0.970275	0.968248
CKSAAGP_GradientBoosting_HashingVectorizer	0.893878	0.931915	0.933014	0.9125	0.858764	0.958115	0.968084	0.965475
CKSAAP_GradientBoosting_HashingVectorizer	0.914286	0.864865	0.910686	0.888889	0.815197	0.908377	0.96037	0.954719
CTDC_GradientBoosting_HashingVectorizer	0.877551	0.934783	0.92823	0.905263	0.848685	0.960733	0.969495	0.968188
CTDD_GradientBoosting_HashingVectorizer	0.906122	0.932773	0.937799	0.919255	0.86893	0.958115	0.969794	0.968624
CTDT_GradientBoosting_HashingVectorizer	0.938776	0.821429	0.896332	0.87619	0.792904	0.86911	0.966717	0.965281
CTriad_GradientBoosting_HashingVectorizer	0.853061	0.945701	0.923445	0.896996	0.839153	0.968586	0.963116	0.955312
DDE_GradientBoosting_HashingVectorizer	0.926531	0.80212	0.881978	0.859848	0.764716	0.853403	0.965306	0.963231
DPC_GradientBoosting_HashingVectorizer	0.902041	0.902041	0.923445	0.902041	0.839214	0.937173	0.96895	0.967348
GAAC_GradientBoosting_HashingVectorizer	0.930612	0.890625	0.92823	0.91018	0.851035	0.926702	0.976034	0.971963
GDPC_GradientBoosting_HashingVectorizer	0.922449	0.849624	0.905901	0.88454	0.807298	0.895288	0.970649	0.965013
GTPC_GradientBoosting_HashingVectorizer	0.934694	0.867424	0.91866	0.899804	0.833151	0.908377	0.973384	0.969532
KSCTriad_GradientBoosting_HashingVectorizer	0.959184	0.743671	0.854864	0.83779	0.729112	0.787958	0.97145	0.97125
TPC_GradientBoosting_HashingVectorizer	0.926531	0.759197	0.856459	0.834559	0.720987	0.811518	0.96147	0.95093

Table_S 7 Performance Metrics and Differences from Baseline after Stepwise Removal of Protein

Descriptors in the GradientBoosting_Word2Vec Model

	Recall	Precision	Accuracy	F1 Score	MCC	Specificity	AUROC	AUPRC
AAC_GradientBoosting_HashingVectorizer	-0.06531	-0.01089	-0.02871	-0.04062	-0.05958	-0.00524	-0.01492	-0.01644
CKSAAGP_GradientBoosting_HashingVectorizer	-0.02449	-0.04211	-0.02552	-0.03288	-0.05427	-0.02618	-0.01711	-0.01922
CKSAAP_GradientBoosting_HashingVectorizer	-0.00408	-0.10916	-0.04785	-0.05649	-0.09783	-0.07592	-0.02482	-0.02997
CTDC_GradientBoosting_HashingVectorizer	-0.04082	-0.03924	-0.0303	-0.04011	-0.06435	-0.02356	-0.0157	-0.0165
CTDD_GradientBoosting_HashingVectorizer	-0.01224	-0.04125	-0.02073	-0.02612	-0.0441	-0.02618	-0.0154	-0.01607
CTDT_GradientBoosting_HashingVectorizer	0.020408	-0.1526	-0.0622	-0.06919	-0.12013	-0.11518	-0.01847	-0.01941
CTriad_GradientBoosting_HashingVectorizer	-0.06531	-0.02832	-0.03509	-0.04838	-0.07388	-0.01571	-0.02208	-0.02938
DDE_GradientBoosting_HashingVectorizer	0.008163	-0.17191	-0.07656	-0.08553	-0.14832	-0.13089	-0.01988	-0.02146
DPC_GradientBoosting_HashingVectorizer	-0.01633	-0.07199	-0.03509	-0.04334	-0.07382	-0.04712	-0.01624	-0.01734
GAAC_GradientBoosting_HashingVectorizer	0.012245	-0.0834	-0.0303	-0.0352	-0.062	-0.05759	-0.00916	-0.01273
GDPC_GradientBoosting_HashingVectorizer	0.004082	-0.1244	-0.05263	-0.06084	-0.10573	-0.08901	-0.01454	-0.01968
GTPC_GradientBoosting_HashingVectorizer	0.016327	-0.1066	-0.03987	-0.04557	-0.07988	-0.07592	-0.01181	-0.01516
KSCTriad_GradientBoosting_HashingVectorizer	0.040816	-0.23036	-0.10367	-0.10759	-0.18392	-0.19634	-0.01374	-0.01344
TPC_GradientBoosting_HashingVectorizer	0.008163	-0.21483	-0.10207	-0.11082	-0.19204	-0.17277	-0.02372	-0.03376

Table_S 8 Performance Analysis of the ScikitRNN_CountVectorizer Model after the Removal of Protein Descriptors

	Recall	Precision	Accuracy	F1 Score	MCC	Specificity	AUROC	AUPRC
AAC_ScikitRNN_CountVectorizer	0.767347	0.427273	0.507177	0.548905	0.114822	0.340314	0.571482	0.463316
CKSAAGP_ScikitRNN_CountVectorizer	0.987755	0.404007	0.425837	0.57346	0.125671	0.065445	0.727514	0.603205
CKSAAP_ScikitRNN_CountVectorizer	0.012245	0.5	0.60925	0.023904	0.022009	0.992147	0.648937	0.531311
CTDC_ScikitRNN_CountVectorizer	0.906122	0.400722	0.433812	0.555695	0.056305	0.13089	0.619334	0.534533
CTDD_ScikitRNN_CountVectorizer	0.963265	0.890566	0.939394	0.92549	0.87646	0.924084	0.98893	0.984607
CTDT_ScikitRNN_CountVectorizer	0.416327	0.733813	0.712919	0.53125	0.375253	0.903141	0.792873	0.681964
CTriad_ScikitRNN_CountVectorizer	0.110204	0.627907	0.626794	0.1875	0.131892	0.958115	0.602265	0.514129
DDE_ScikitRNN_CountVectorizer	0.881633	0.417795	0.473684	0.566929	0.12017	0.212042	0.6266	0.530496
DPC_ScikitRNN_CountVectorizer	0.893878	0.438	0.510367	0.587919	0.19215	0.264398	0.712801	0.616047
GAAC_ScikitRNN_CountVectorizer	0.032653	0.666667	0.61563	0.062257	0.078992	0.989529	0.646543	0.525518
GDPC_ScikitRNN_CountVectorizer	0.012245	1	0.614035	0.024194	0.08658	1	0.774239	0.713242
GTPC_ScikitRNN_CountVectorizer	0.4	0.532609	0.628389	0.456876	0.187377	0.774869	0.653489	0.552128
KSCTriad_ScikitRNN_CountVectorizer	0	0	0.60925	0	0	1	0.580003	0.448171
TPC_ScikitRNN_CountVectorizer	0.085714	0.7	0.628389	0.152727	0.142081	0.97644	0.665595	0.585732

Table_S 9 Performance Metrics and Differences from Baseline after Stepwise Removal of Protein Descriptors in the ScikitRNN_CountVectorizer Model

	Recall	Precision	Accuracy	F1 Score	MCC	Specificity	AUROC	AUPRC
AAC_ScikitRNN_CountVectorizer	-0.20408	0.039651	0.118022	-0.00523	0.158873	0.324607	-0.18958	-0.23979
CKSAAGP_ScikitRNN_CountVectorizer	0.016327	0.016385	0.036683	0.019327	0.169722	0.049738	-0.03355	-0.0999
CKSAAP_ScikitRNN_CountVectorizer	-0.95918	0.112378	0.220096	-0.53023	0.06606	0.97644	-0.11213	-0.17179
CTDC_ScikitRNN_CountVectorizer	-0.06531	0.0131	0.044657	0.001562	0.100356	0.115183	-0.14173	-0.16857
CTDD_ScikitRNN_CountVectorizer	-0.00816	0.502944	0.550239	0.371357	0.920511	0.908377	0.227866	0.281505
CTDT_ScikitRNN_CountVectorizer	-0.5551	0.346191	0.323764	-0.02288	0.419304	0.887435	0.031809	-0.02114

CTriad_ScikitRNN_CountVectorizer	-0.86122	0.240285	0.23764	-0.36663	0.175943	0.942408	-0.1588	-0.18897
DDE_ScikitRNN_CountVectorizer	-0.0898	0.030173	0.08453	0.012796	0.164221	0.196335	-0.13446	-0.17261
DPC_ScikitRNN_CountVectorizer	-0.07755	0.050378	0.121212	0.033787	0.236201	0.248691	-0.04826	-0.08706
GAAC_ScikitRNN_CountVectorizer	-0.93878	0.279045	0.226475	-0.49188	0.123043	0.973822	-0.11452	-0.17758
GDPC_ScikitRNN_CountVectorizer	-0.95918	0.612378	0.22488	-0.52994	0.130631	0.984293	0.013174	0.01014
GTPC_ScikitRNN_CountVectorizer	-0.57143	0.144987	0.239234	-0.09726	0.231428	0.759162	-0.10758	-0.15097
KSCTriad_ScikitRNN_CountVectorizer	-0.97143	-0.38762	0.220096	-0.55413	0.044051	0.984293	-0.18106	-0.25493
TPC_ScikitRNN_CountVectorizer	-0.88571	0.312378	0.239234	-0.40141	0.186132	0.960733	-0.09547	-0.11737

Table_S 10 Comparative Performance Analysis of the RandomForest_Doc2Vec Model after Simultaneous Removal of AAC and GAAC Protein Descriptors

	Recall	Precision	Accuracy	F1 Score	MCC	Specificity	AUROC	AUPRC
AAC_GAAC_RandomForest_Doc2Vec	0.853061224	0.88185654	0.897926635	0.867219917	0.784640565	0.926701571	0.936991132	0.92966836
AAC_RandomForest_Doc2Vec	0.857142857	0.9375	0.92185008	0.895522388	0.835436435	0.963350785	0.958756277	0.952987488
GAAC_RandomForest_Doc2Vec	0.865306122	0.909871245	0.913875598	0.887029289	0.818185546	0.945026178	0.957217651	0.953268706
RandomForest_Word2Vec	0.734694	0.947368	0.880383	0.827586	0.752223	0.973822	0.935271	0.930686

Table_S 11 Comparative Performance Analysis of the ScikitRNN_CountVectorizer Model after Simultaneous Removal of CTDD and DPC Protein Descriptors

	Recall	Precision	Accuracy	F1 Score	MCC	Specificity	AUROC	AUPRC
CTDD_DPC_ScikitRNN_CountVectorizer	0.951020408	0.872659176	0.926634769	0.91015625	0.850594857	0.910994764	0.986611818	0.981679454
CTDD_ScikitRNN_CountVectorizer	0.963265306	0.890566038	0.939393939	0.925490196	0.876459803	0.92408377	0.988930441	0.984606993
DPC_ScikitRNN_CountVectorizer	0.893877551	0.438	0.510366826	0.587919463	0.192150464	0.264397906	0.712800513	0.616047143
ScikitRNN_CountVectorizer	0.971429	0.387622	0.389155	0.554133	-0.04405	0.015707	0.761064	0.703102

Table_S 12 Comparative Performance Analysis of Different Models

	Recall	Precision	Accuracy	F1 Score	MCC	Specificity	AUROC	AUPRC
GAAC_Bagging_Doc2Vec	0.62449	0.974522	0.84689	0.761194	0.691508	0.989529	0.949738	0.933288
AAC_RandomForest_Doc2Vec	0.857142857	0.9375	0.92185008	0.895522388	0.835436435	0.963350785	0.958756277	0.952987488
GradientBoosting_HashingVectorizer	0.918367347	0.974025974	0.958532695	0.945378151	0.913031158	0.984293194	0.985190726	0.984690651
CTDD_ScikitRNN_CountVectorizer	0.963265	0.890566	0.939394	0.92549	0.87646	0.924084	0.98893	0.984607

Table_S 13 Comparison of Data Standardization and Oversampling Results

	Recall	Precision	Accuracy	F1 Score	MCC	Specificity	AUROC	AUPRC
MinMaxScaler_GradientBoosting_HashingVectorizer	0.938776	0.864662	0.91866	0.900196	0.833754	0.905759	0.976301	0.972332
StandardScaler_GradientBoosting_HashingVectorizer	0.808163	0.985075	0.920255	0.887892	0.836701	0.992147	0.974976	0.971103
smote_GradientBoosting_HashingVectorizer	0.922449	0.961702	0.955343	0.941667	0.906032	0.97644	0.982178	0.982439
Cost-sensitive_GradientBoosting_HashingVectorizer	0.959184	0.70997	0.830941	0.815972	0.691849	0.748691	0.971974	0.966414

Table_S 14 Analysis of the Impact of Different Cross-Validation Iterations on Model Performance

	Recall	Precision	Accuracy	F1 Score	MCC	Specificity	AUROC	AUPRC
tenFold_GradientBoosting_HashingVectorizer	0.959184	0.79932	0.889952	0.871985	0.786812	0.84555	0.971001	0.964396
twoFold_GradientBoosting_HashingVectorizer	0.918367	0.882353	0.920255	0.9	0.834192	0.921466	0.971546	0.969254
fiveFold_GradientBoosting_HashingVectorizer	0.897959	0.940171	0.937799	0.91858	0.8689	0.963351	0.977102	0.97482

Table_S 15 Analysis of the Impact of Different Feature Selection Methods on Model Performance

	Recall	Precision	Accuracy	F1 Score	MCC	Specificity	AUROC	AUPRC
Chi2_GradientBoosting_HashingVectorizer	0.053061	0.866667	0.626794	0.1	0.152705	0.994764	0.874004	0.784015
f_classif_GradientBoosting_HashingVectorizer	0.963265	0.877323	0.933014	0.918288	0.864443	0.913613	0.976087	0.974432
Mutual Information_GradientBoosting_HashingVectorizer	0.906122	0.948718	0.944179	0.926931	0.882417	0.968586	0.970157	0.967931
Variance Threshold_GradientBoosting_HashingVectorizer	0.428571	0.444915	0.567783	0.43659	0.086247	0.657068	0.563703	0.476225

Table_S 16 Cumulative Analysis of Feature Weights for Different Protein Descriptors

feature_name	Importance_sum
AAC	0.08287
CKSAAGP	0.004466
CKSAAP	0.036844
CTDC	0.036797
CTDD	0.002001
CTDT	0.006275
CTriad	0.001475
DDE	0.05283
DPC	0.014825
GAAC	0.23415
GDPC	0.001714
GTPC	0.001655
KSCTriad	0.000633
TPC	0.036106
str_feature	0.48736

Table_S 17 Ranking of the Top 30 Sub-Feature Importances

	feature_name	Feature	Importance
0	str_feature	feature_599	0.302314
1	GAAC	uncharge	0.23415
2	str_feature	feature_59	0.076174
3	AAC	P	0.041766
4	str_feature	feature_76	0.032335
5	str_feature	feature_781	0.030272
6	AAC	I	0.025993
7	CTDC	hydrophobicity_CASG920101.G3	0.017529
8	str_feature	feature_0	0.016891
9	str_feature	feature_644	0.015942
10	DDE	EQ.1	0.015675
11	CTDC	charge.G1	0.011063
12	str_feature	feature_17	0.008954
13	DDE	QQ.1	0.008557

14	CKSAAP	EE.gap0	0.007625
15	DDE	EG.1	0.007484
16	DDE	QL.1	0.007311
17	DPC	QL	0.005718
18	CTDT	charge.Tr1221	0.005627
19	DPC	PE	0.004995
20	str_feature	feature_45	0.004312
21	AAC	D	0.003952
22	CKSAAP	LE.gap0	0.003559
23	AAC	E	0.003506
24	CKSAAP	KE.gap0	0.002927
25	CTDC	hydrophobicity_ZIMJ680101.G2	0.002744
26	AAC	H	0.002281
27	CKSAAP	EQ.gap0	0.0022
28	CKSAAGP	uncharger.uncharger.gap4	0.002004
29	TPC	KDL	0.001864
30	DDE	EE.1	0.001714
